# Resolution of Thyrotoxicosis-Associated Pulmonary Hypertension With Treatment of Thyrotoxicosis: A Case-Based Report and Literature Review

**DOI:** 10.7759/cureus.21304

**Published:** 2022-01-16

**Authors:** Zahid Khan, Gideon Mlawa, Mahmood Bashir, Mohammed Abumedian, Saeed Yousif, Animesh Gupta, Vinod Warrier

**Affiliations:** 1 Cardiology, Royal Free Hospital, London, GBR; 2 Internal Medicine and Diabetes and Endocrinology, Barking, Havering and Redbridge University Hospitals NHS Trust, London, GBR; 3 Internal Medicine, Barking, Havering and Redbridge University Hospitals NHS Trust, London, GBR; 4 Geriatrics, Barking, Havering and Redbridge University Hospitals NHS Trust, London, GBR; 5 Acute Internal Medicine, Barking, Havering and Redbridge University Hospitals NHS Trust, Romford Essex, GBR; 6 Internal Medicine, Mid and South Essex NHS Foundation Trust, Southend on Sea, GBR

**Keywords:** grave's disease, medications non-compliance, non-compliance to self-management, heart failure with preserved ejection fraction, t3-thyrotoxicosis, – pulmonary hypertension

## Abstract

We present a case of a 41-year-old Afro-Caribbean female, who was diagnosed with thyrotoxic Graves’ disease. She had a past medical history of hypertension and was on amlodipine and valsartan. There was no significant family history of note. She initially presented to Emergency Department with palpitations and excessive sweating. Her thyroid-stimulating hormone was <0.02mu/L and free triiodothyronine (T3) 29.5pmol/L at the time of diagnosis. The thyroid peroxidase antibody test was negative. She was started on carbimazole 15mg once daily and propranolol 40mg twice daily. She remained non-compliant to treatment for over two years and missed most outpatient clinic appointments and her condition remained poorly controlled during this time period. She was re-admitted to the hospital after 18 months, with high output congestive cardiac failure. An echocardiogram showed pulmonary hypertension and her right ventricular systolic pressure was measured to be 70-75mmHg. She was started on Lugol's iodine 0.2mls three times daily, propranolol 40mg three times daily, cholestyramine 4 gram four times a day, propylthiouracil 100mg four times a day. After 3 weeks of treatment, she became euthyroid and her pulmonary hypertension improved dramatically with treatment. She underwent total thyroidectomy after a few weeks and biopsies confirmed the findings of Graves’ disease.

## Introduction

Pulmonary hypertension (PHT) is defined as mean pulmonary artery pressure (PAP) of more than 25mmHg at rest, pulmonary artery wedge pressure less than 15mmHg, and >30mmHg with exercise [1]. The prevalence of PHT is approximately 5-50 per one million adults and the serious life-threatening disease is two to four times more common in women compared to men [2,3].

Hyperthyroidism can present with various cardiac features such as cardiomegaly, increased cardiac output, atrial fibrillation (AF), and, in certain cases, congestive heart failure [4]. Recently, there has been growing interest to explore the association between hyperthyroidism and PHT again, although the association was first described in 1980 [3]. Thyrotoxicosis is associated with both right and left ventricular dysfunction in classic thyrotoxic cardiomyopathy and it should be considered in all cases of right ventricular dysfunction that can not be explained by other causes [5]. Thyrotoxicosis has profound cardiovascular effects, however, only about 6% of patients presented with cardiomyopathy as initial clinical presentation. From this 6%, less than 1% develop impaired left ventricular systolic function with dilated cardiomyopathy that mostly responded to conventional treatment [6,7].

The mechanism of PHT is multi-factorial, however, the most likely explanation is the increased blood volume in patients with hyperthyroidism, decreased peripheral vascular resistance, increased resting heart rate and left ventricular (LV) contractility, which then results in a hyperdynamic circulatory state [6]. Sinus tachycardia is common in patients with thyrotoxicosis, although AF is found in 10-25% of patients [8,9].

## Case presentation

A 41-year-old Afro-Caribbean female was diagnosed with thyrotoxic Graves’ disease. Her only relevant past medical history was hypertension, for which she was on amlodipine and valsartan. There was no family history of thyroid disease. Prior to admission, she has been complaining of generalized fatigue and occasional palpitations. She did not have any other cardiac risk factor apart from hypertension and she was a lifelong non-smoker and non-drinker. Her initial blood tests showed a very low thyroid-stimulating hormone (TSH) <0.001 mu/L, free triiodothyronine (FT3) 29.5pmol/L and she had a negative thyroid peroxidase (TPO) antibody test. Her other autoimmune screen was negative. Subsequently, she was started on carbimazole 15mg once daily (OD) and propranolol 40mg twice daily (BD).

She was non-compliant with treatment and her hyperthyroidism remained poorly controlled for over 2 years. During this time, she failed to attend clinic appointments. Although she was non-compliant with treatment, she managed to have regular blood tests, which showed clinical deterioration. Her lab tests two months after the initial discharge showed TSH < 0.0001, FT3 39.3pmol/L, FT3 47.8pmol/L at 12 months, and FT3 level of 50pmol/L at 16 months after the initial discharge.

**Figure 1:**
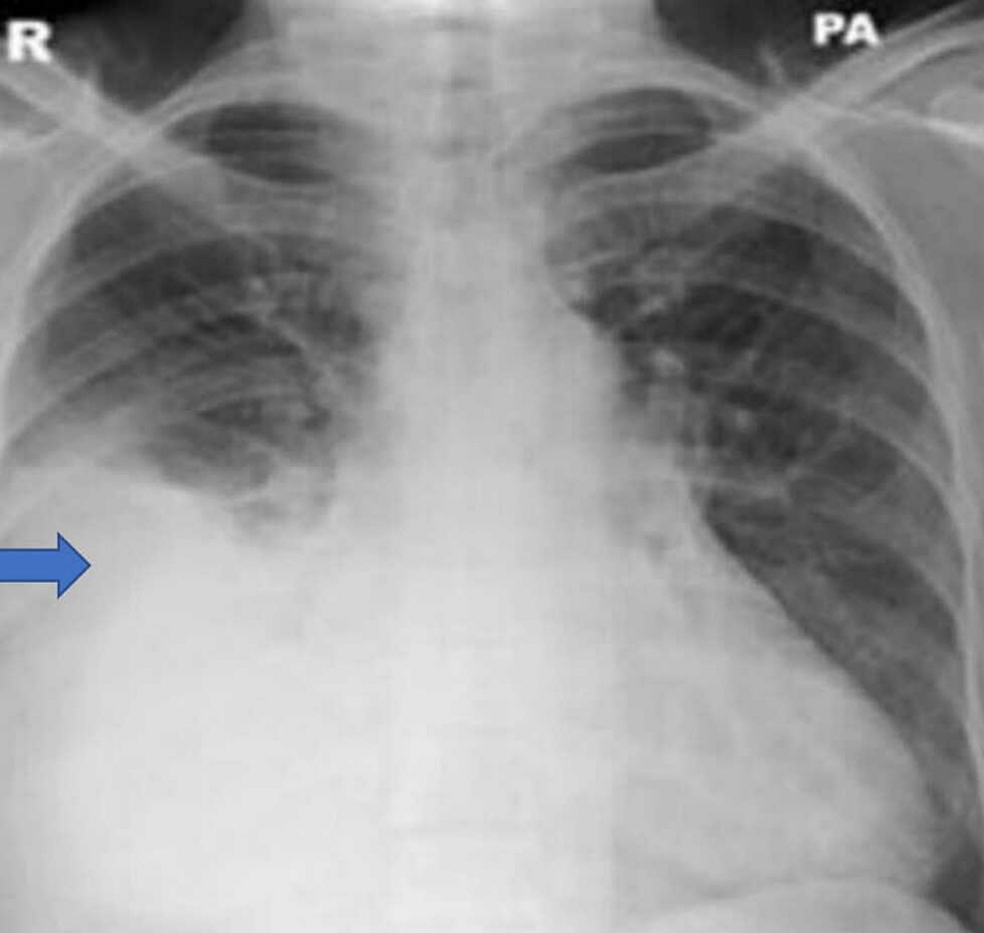
Chest radiograph showing right-sided pleural effusion

She was readmitted to the hospital 18 months later with high output congestive cardiac failure with left ventricular ejection fraction (LVEF) about 50%. She had peripheral oedema to the groin and jugular venous pressure (JVP) was > 8cm. Lab tests showed TSH <0.02mu/L, FT3 50pmol/L, free thyroxine (FT4) 99.9pmol/L. Chest radiography showed a right-sided pleural effusion (Figure 1).

Echocardiogram (Echo) showed right ventricular systolic pressure 70-75mmHg consistent with pulmonary hypertension, moderate tricuspid regurgitation, but normal left ventricular function and size. Thyroid scan showed both glands including the isthmus to be diffusely enlarged with heterogeneous echogenicity. There was no retrosternal extension present on either side. Colour doppler examination showed features of thyroid inferno, consistent with Graves' disease.

She was started on Lugol`s iodine 0.2mls times daily (tds), propranolol 40mg tds, cholestyramine 4 gram four times daily (qds), propylthiouracil 100mg qds, spironolactone 50mg od and treatment doses of tinzaparin 11,000 units/daily. 

After 3 weeks, she became euthyroid and her lab tests showed TSH<0.02mu/L, FT3 4.0pmol/L, FT4 13.6pmol/L) (Table 1).

**Table 1:** Thyroid function test results trend with treatment TSH: thyroid-stimulating hormone; T3: triiodothyronine; T4: thyroxine

DATE	DAY	TSH (0.5 to 5.0 mIU/L)	FREE T3 (2.8 – 7.1 pmol/L)	FREE T4 (12-22 pmol/L))	TREATMENT
29^th^ May	0	<0.02	>50	>99.9	Propylthiouracil Propranolol Lugol’s Iodine Cholestyramine
31^st^ May	2	<0.02	15.5	47.1	Propylthiouracil Propranolol Lugol’s Iodine Cholestyramine
3^rd^ June	5	<0.02	8.5	26.9	Cholestyramine stopped
8^th^ June	10	<0.02	4.0	13.6	Thyroxine started in block and replace regimen
13^th^ June	15	<0.02	2.4	4.7	Lugol’s iodine and propranolol stopped
20^th^ June	22	<0.02	3.5	9.8	Propylthiouracil Thyroxine
24^th^ June	26	Thyroidectomy			Propylthiouracil stopped

She also showed significant clinical improvement and her PHT improved dramatically on echocardiography and right ventricular systolic pressure dropped to 36-41mmHg (Table 2).

**Table 2:** Echocardiography pulmonary hypertension and tricuspid regurgitation values pre and post treatment RVSP: right ventricular systolic pressure; TR: tricuspid regurgitation

ECHO FINDINGS	31^ST^ MAY	23^RD^ JUNE
Pulmonary hypertension	At least moderate	Mild
RVSP (15-25 mmHg)	70-75	36-41
Peak TR gradient (35-36 mmHg)	60	31

**Figure 2:**
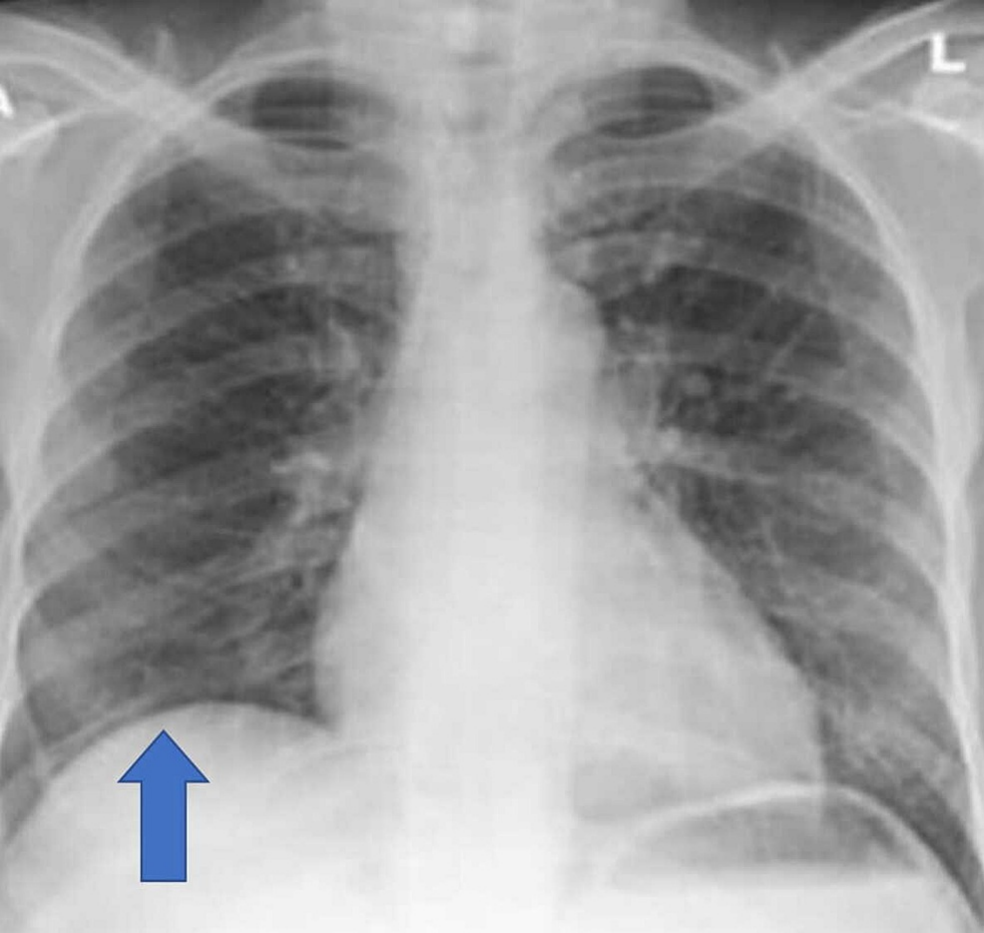
Chest radiograph shows resolution of the right-sided pleural effusion

Repeat chest radiography showed resolution of the right-sided pleural effusion (Figure 2).

After 6 weeks of treatment, she underwent a total thyroidectomy. Biopsy findings were compatible with treated Graves’ disease. She also had thyroid radioactive iodine uptake scan (RAIU) uptake that showed iodine uptake >40% after 24 hours. She had further follow-up in the outpatient clinic and has remained stable on treatment. Her latest echocardiogram showed complete resolution of the pulmonary hypertension and her exercise tolerance has improved significantly.

## Discussion

Pulmonary hypertension (PHT) is a sustained elevation of pulmonary arterial pressure to over 25mmHg at rest or over 30mmHg with exercise. PHT is classified as primary (idiopathic) or secondary. The prevalence of PHT is 0.13% and primary PHT is found at autopsy in about 1% of patients with cor pulmonale [2,4,7]. Several case reports published in the past have reported patients with hyperthyroidism presenting as right heart failure and tricuspid regurgitation. A study of 23 consecutive patients with hyperthyroidism caused by Graves’ disease, showed that 65% of patients had pulmonary hypertension which resolved on adequate treatment of the underlying Graves' disease [10,11].

It is also important to mention that both severe and chronic hyperthyroidism are associated with sinus tachycardia and atrial arrhythmias such as atrial fibrillation or flutter, and if left untreated, exaggerated sinus tachycardia or atrial fibrillation can produce rate-related left ventricular dysfunction and heart failure [12]. Patients with ischaemic heart disease (IHD) or hypertensive heart disease (HHD) are predisposed to the development of heart failure [12]. In addition, both Graves’ and Hashimoto’s diseases are also found to be associated with a higher prevalence of mitral valve prolapse, which predisposes patients to left atrial enlargement and atrial fibrillation [13]. Chu et al (2002) found 49% of patients with primary PHT had a diagnosis of thyroid disease clinically, biochemically, and serologically [7]. The postulated mechanism of tachycardia is through excessive beta-adrenergic activity due to an increased number of beta-adrenergic receptors in hyperthyroidism

Armigliato et al (2006) assessed the pulmonary artery systolic pressure (PASP) in 23 consecutive patients diagnosed with hyperthyroidism due to Graves' disease and half of them (52%) did not show antithyroglobulin and antithyroperoxidase antibodies [14]. Four patients from this cohort were lost to follow-up; they were able to evaluate 17 patients serially with echocardiography and 16 patients normalized their PASP value. From this group, 13 patients achieved normal PASP values after methimazole, two after total thyroidectomy, and one after (131)I treatment.

Siu et al (2006), reported that there were no significant differences in the clinical characteristics of hyperthyroid patients with or without PHT, although, patients with PHT had significantly higher cardiac output (CO), PASP, peak transmitral early diastolic flow velocity (E), and the ratio of E to early diastolic mitral annular velocity (E') compared with those without PHT [15]. The patient, in this case, had PASP > 75mmHg and inferior vena cava (IVC) diameter was 2.4cm with < 50% collapsibility on inspiration. The pulmonary acceleration time was < 60ms in this patient. Similarly, another study reported the restoration of normal PASP and resolution of PHT in a 43-year-old patient with severe pulmonary hypertension, moderate to severe tricuspid regurgitation, normal left heart function and a negative bubble contrast study after 11 months of treatment [16]. The use of beta-blockade was previously considered to be contraindicated in patients with PHT, however, over the last few years, these guidelines have changed and the use of beta-blockers is recommended to rate control these patients [17]. Although the definitive treatment remains radioactive Iodine, however, the use of diuretics and digoxin is appropriate in these patients [18,19].

## Conclusions

Thyrotoxicosis may present with features of pulmonary hypertension, especially in patients with poor compliance. This major clinical complication should be managed by rapid effective treatment of hyperthyroid state as inpatient and temporary anticoagulation if there is evidence of atrial arrhythmia such as atrial fibrillation. Hyperthyroidism should always be considered in the differential diagnosis of patients presenting with pulmonary hypertension.

Pulmonary hypertension due to hyperthyroidism is reversible and the use of beta-blockade and digoxin is appropriate in these patients. The most effective treatment remains radioactive iodine for these patients. Dilated cardiomyopathy secondary to hyperthyroidism shows significant recovery and resolution of pulmonary hypertension by achieving a euthyroid state.
